# Elucidating the effect of deferoxamine, a hypoxia mimetic agent, on angiogenesis restoration in endothelial progenitor cells (EPCs) from diabetic mice

**DOI:** 10.22038/ijbms.2025.81969.17737

**Published:** 2025

**Authors:** Vahid Siavashi, Seyed Mahdi Nassiri, Mahdi Farhadi Mahalli, Tunku Kamarul, Ali Mohammad Sharifi

**Affiliations:** 1 Department of Pharmacology and Razi Drug Research Center, School of Medicine, Iran University of Medical Sciences, Tehran, Iran; 2 Department of Clinical Pathology, Faculty of Veterinary Medicine, University of Tehran, Tehran, Iran; 3 Department of Biology, Science and Research Branch, Islamic Azad University, Tehran, Iran; 4 Tissue Engineering Group (NOCERAL), Department of Orthopedic Surgery, Faculty of Medicine, University of Malaya, Kuala Lumpur, Malaysia; 5 Department of Pharmacology, School of Medicine, Iran University of Medical Sciences, Tehran, Iran; 6 Stem cell and Regenerative Medicine Research Center, Iran University of Medical Sciences, Tehran, Iran

**Keywords:** Deferoxamine, Diabetes, Endothelial progenitor cell HIF-α, Tie2, VEGF

## Abstract

**Objective(s):**

Diabetes increases the risk of heart disease and stroke, primarily through endothelial cell dysfunction and vascular damage. These vascular complications are partly due to defects in endothelial progenitor cells (EPCs). This study explores the efficacy of pharmacological priming of bone marrow EPCs (BMEPCs) with Deferoxamine (DFO), a hypoxia mimetic agent, in restoring dysregulated angiogenic pathways in streptozotocin (STZ)-induced mice with type-1 diabetes (T1D).

**Materials and Methods:**

BMEPCs were isolated from both normal and STZ-induced mice with T1D. The effects of an optimal concentration of DFO (80 µM) on the viability, proliferation, and tubulogenesis of EPCs were assessed. Furthermore, the probable beneficial effects of the conditioned medium from EPCs treated in the presence and absence of DFO were examined in mice (T1D) wound healing models.

**Results:**

DFO (80 µM) increased cell viability, proliferation, and tubulogenesis. EPCs isolated from diabetic mice showed significant impairments in the expression of HIF-1α, VEGF, and SDF-1 proteins compared to controls. DFO-preconditioning significantly enhanced protein expression of these genes. The conditioned medium from diabetic EPCs treated with DFO had a substantially greater favorable effect on wound healing in diabetic mice, connected with elevated levels of HIF-1α, VEGF, phosphorylated Tie2/Tie2, and Ang1.

**Conclusion:**

DFO reactivates proliferation and restores the impaired angiogenic properties of EPCs from diabetic mice by stabilizing HIF-1α and VEGF. Additionally, DFO enhanced the pro-angiogenic activity in the EPC-secretome, leading to improved wound healing. This improvement is attributed to the dual activation of HIF-1α /VEGF and Ang-1/Tie2 pathways, which are crucial for initiating and maturing new blood vessels.

## Introduction

Diabetes mellitus (DM) is one of the most serious health problems worldwide. DM is a complex metabolic disorder characterized by hyperglycemia due to impaired insulin secretion or resistance, often leading to slow or limited wound healing. One of the most common complications in diabetic patients is impaired angiogenesis (1). Angiogenesis, essential for development, wound healing, and organ homeostasis (2), is regulated by several key factors, including vascular endothelial growth factor (VEGF)(3), hypoxia-inducible factor-1 (HIF-1)(4), angiopoietin-1 (Ang-1), angiopoietin-2 (Ang-2)(Chen and Stinnett, 2008) (5), and stromal cell-derived factor-1 (SDF-1) (6). Therapeutic angiogenesis, using cell and pharmacotherapy, is considered an efficient strategy to restore normal circulation in many diabetic patients. However, there is strong evidence that the function of cells involved in angiogenesis is impaired in diabetics (7-9).

Endothelial progenitor cells (EPCs) are derived from bone marrow and mobilized to peripheral blood to stimulate compensatory angiogenesis (10). These cells express surface markers similar to vascular endothelial cells, adhere to the endothelium, and participate in neoangiogenesis (11). EPCs are characterized by partial expression of CD133, Tie-2, and VEGF receptor 2 (KDR) (10, 12). They maintain vascular homeostasis and mediate vascular repair under both pathological and physiological conditions (13, 14). Previous studies have shown reduced mobilization and function of EPCs in type 1 and type 2 diabetic patients (15). Recently, various strategies have been proposed to enhance the functional capacity of impaired stem cells, including preconditioning with hypoxic shock (16) and pharmacological agents that mimic hypoxia (17-19).

Deferoxamine (DFO), an iron-chelating agent, has shown promise in up-regulating VEGF in ischemic flap surgery (20) by stabilizing HIF-1 protein in mesenchymal stem cells (18, 21). HIF-1α, a subunit of HIF-1, is a key transcription factor necessary for the expression of angiogenic growth factors like VEGF and for the recruitment of EPCs to ischemic sites to form new blood vessels (22, 21). Under normoxic conditions, HIF-1α is hydroxylated by prolyl hydroxylases, which target it for degradation. However, DFO stabilizes HIF-1α by chelating iron, a cofactor for prolyl hydroxylases, thereby preventing its degradation and promoting the transcription of HIF-1α target genes. This stabilization of HIF-1α under hypoxic conditions leads to enhanced angiogenic responses, even in the diabetic state (19).

The interplay between HIF-1, VEGF, and inflammatory cytokines like Tumor Necrosis Factor-alpha (TNF-α) and Interleukin-10 (IL-10) is crucial in angiogenesis. HIF-1 regulates VEGF expression under hypoxic conditions, but its activity is impaired in diabetic conditions. TNF-α inhibits EPC proliferation and survival, while IL-10 promotes EPC viability and angiogenesis, highlighting the complex inflammatory milieu in diabetes. IL-10, as an anti-inflammatory cytokine primarily signals via STAT3, has been indicated to drive EPC recruitment to injured tissues (23).

Additionally, the Tie-2/Angiopoietin signaling pathway plays a crucial regulatory role in angiogenesis. Tie-2, a receptor tyrosine kinase expressed on endothelial cells, interacts with its ligand angiopoietin-1 (Ang-1) and angiopoietin-2 (Ang-2). Ang-1 promotes vessel maturation and stabilization, maintaining vascular quiescence and integrity, while Ang-2 can act as an antagonist or agonist of Tie-2, depending on the context, promoting vessel sprouting in the presence of VEGF or causing vessel regression in its absence. This signaling axis is essential for proper vascular remodeling and the transition from nascent to mature vascular networks (24).

This study aims to investigate the efficacy of DFO in restoring angiogenic function in EPCs derived from STZ-diabetic mice. By elucidating the interplay between HIF-1 impairment, inflammatory cytokine dynamics (IL-10 and TNF-α), the Tie-2/Angiopoietin signaling pathway, and the angiogenic capacity of diabetic EPCs, we seek to uncover novel therapeutic strategies to enhance vascular repair in diabetic conditions. This study aimed to investigate the biological features of EPCs primed with DFO in diabetic and non-diabetic mice. We sought to evaluate the effects of DFO preconditioning for promoting the angiogenic potential of EPCs to enhance the efficacy of cell therapy for wound healing in DM.

Our findings could also provide critical insights into the potential of hypoxia mimetics as a viable approach to counteract the vascular complications associated with diabetes.

## Materials and Methods

### Antibodies and reagents

The following reagents and antibodies were utilized in this study: APC-labeled rat anti-mouse CD133/AC133 (Cat No: 17-1331, eBioscience, USA), PE-labeled rat anti-mouse Tie2 (Cat No: 12-5987-81, eBioscience, USA), and Pacific Blue-conjugated rat anti-mouse VEGFR2 (Cat No: 121914, BioLegend, USA). Additional PE-conjugated antibodies included anti-mouse CD45 (number: 12-0451-81, eBioscience), CD14 (Cat No: 123309, BioLegend), CD11b (Cat No: 12-0112-81, eBioscience), and CD105 (Cat No: 120407, BioLegend).

For immunofluorescence, goat anti-rabbit secondary antibody conjugated to Texas Red (1:1000, Cat No: ab6787, Abcam) and an anti-Ki67 primary antibody (Catalog number: ab15580, Abcam) were used. Nuclear staining was performed using DAPI (1 µg/ml, Cat No: D9542, Sigma-Aldrich).

Cell culture reagents included fibronectin (1 µg/ml, Cat No: C-43050) and EGM-2 Supplement Pack (Cat No: C-39211), both from PromoCell (Germany), as well as Ficoll (Cat No: F5415, Sigma) for density gradient separation. Endothelial function was assessed using DiI-acetylated LDL (Cat No: L-3484, Thermo Fisher) and Matrigel (Cat No: 354230, Corning) for angiogenesis assays.

Western blot analysis employed PVDF membranes (Cat No: 162-01777, Bio-Rad, USA), blocking with non-fat dry milk (Cat No: 1.15363.0500, Merck, Germany), and HRP-conjugated secondary antibodies: sheep anti-rabbit IgG (1:5000, Catalog number ab6795, Abcam) and rabbit anti-mouse IgG (1:4000, Cat No: ab6728, Abcam). Primary antibodies used included anti-β-actin (Cat No: ab8224), anti-Tie2 (Cat No: ab24859), anti-phospho-Tie2 (Tyr992, Cat No ab78142), and mouse monoclonal anti-HIF-1α (1:1000, Cat No: ab113642), all from Abcam.

A hypoxia-inducible factor (HIF) pathway inhibitor was sourced from Santa Cruz Biotechnology (San Jose, CA, USA). For chromogenic detection, 3,3′-diaminobenzidine (DAB, Cat No: D5637, Sigma-Aldrich) was used. BSA solution (Cat No: A2058, Sigma) served as a blocking agent where applicable.

### Animal

This study was conducted using male C57BL/6 mice. All animal procedures were performed in compliance with the guidelines outlined in the Guide for the Care and Use of Laboratory Animals. The experimental protocol received ethical approval from the Animal Care and Use Committee of Iran University of Medical Sciences.

### MTT assay

DFO was prepared according to the method outlined by Mehrabani *et al*. (19). To assess its effect on EPCs, various concentrations of DFO (20, 40, 80, and 160 µM) were applied. Cell viability was determined using the MTT assay. Briefly, EPCs isolated from mice (1×10⁴ cells per well) were exposed to the indicated DFO concentrations and incubated at 37 ^°^C for either 24 or 48 hr. After treatment, the culture medium was removed, and cells were washed before being incubated for four hours with MTT reagent [3-(4,5-dimethylthiazol-2-yl)-2,5-diphenyltetrazolium bromide].

Following MTT incubation, 100 μl of dimethyl sulfoxide (DMSO) was added to each well to solubilize the formazan crystals, and the contents were mixed thoroughly by pipetting. Absorbance was then measured at 570 nm using a microplate reader. The percentage of viable cells was calculated using the formula:

Cell viability (%) = (OD of treated sample/OD of control) × 100.

### Diabetes induction

To induce diabetes, C57BL/6 mice received a single intraperitoneal injection of streptozotocin (STZ) at a dose of 240 mg/kg, as previously described (25). Blood glucose levels were assessed one week post-injection using a clinical chemistry analyzer (Selectra Pro M). Mice exhibiting glucose levels exceeding 300 mg/dL were selected for further experimentation.

### Isolation, expansion, and cell culture

Following anesthesia with intramuscular injections of ketamine (100 mg/kg) and xylazine (10 mg/kg), cervical dislocation was performed. Femurs from both diabetic and non-diabetic mice were flushed with phosphate-buffered saline (PBS), and the collected cells were passed through a sterile 100 µm cell strainer. The resulting single-cell suspension was layered onto Ficoll and centrifuged at 400× g for 20 minutes to isolate mononuclear cells (MNCs). These MNCs were seeded on fibronectin-coated culture dishes and maintained in M199 medium supplemented with the EGM-2 kit, which contains fetal calf serum (FCS), insulin-like growth factor (IGF), vascular endothelial growth factor (VEGF), basic fibroblast growth factor (bFGF), hydrocortisone, ascorbic acid, heparin, and epidermal growth factor (EGF). Cultures were incubated at 37 ^°^C with 5% CO₂.

### Treatment protocol

EPCs were cultured for seven days in M199 medium supplemented with EGM-2, either in the presence or absence of DFO at 80 µM. To investigate the involvement of HIF-1α in DFO-mediated effects, cells were pre-treated with a HIF pathway inhibitor for one hour prior to DFO exposure.

### Flow cytometry analysis

To perform flow cytometric analysis, 1×10⁶ cells were first incubated with 3% bovine serum albumin (BSA) to block non-specific binding. Cells were then labelled with a set of fluorescently conjugated antibodies: APC-labeled rat anti-mouse CD133/AC133, PE-labeled rat anti-mouse Tie2, and Pacific Blue-labeled rat anti-mouse VEGFR2. Staining was carried out at 4 ^°^C for 30 minutes in the dark. Appropriate isotype controls were included for each antibody to ensure specificity. Following staining, samples were processed using a BD FACSAria II flow cytometer (BD Biosciences), and data analysis was conducted with FlowJo software (version 7.6.5). CD133⁺ cells were gated electronically, and the percentage of cells co-expressing Tie2 and VEGFR2 within this population was quantified.

### EPC characterization

EPCs were characterized according to protocols previously reported by Jabarpour *et al*. (14). Flow cytometry was used to assess surface marker expression using a panel of PE-conjugated antibodies specific for mouse CD45, CD14, CD11b, and CD105. To further verify the identity of EPCs and distinguish them from hematopoietic progenitors, 4×10⁴ cells were cultured in a methylcellulose-based semi-solid medium supplemented with 100 ng/ml vascular endothelial growth factor (VEGF) and 5 ng/ml granulocyte-macrophage colony-stimulating factor (GM-CSF). After two weeks of incubation, colony formation was evaluated, and colonies were counted under a microscope, following the methodology of Keshavarz *et al*. (26).

### Impact of deferoxamine on EPC colony formation

The EPC colony-forming assay was conducted following adapted protocols from previous studies (10, 27, 28). In short, 2×10⁵ mononuclear cells (MNCs) were plated onto fibronectin-coated culture dishes and maintained in M199 medium supplemented with the EGM-2 kit, either in the presence or absence of 80 µM DFO. 

### In vitro tube formation assay (matrigel assay)

To assess the angiogenic potential of cells, an *in vitro* tube formation assay was conducted using growth factor-reduced Matrigel. A total of 2×10⁴ cells were plated per well on 100 μl of Matrigel and incubated at 37 ^°^C for 24 hr. After incubation, tube-like structures were visualized using an inverted microscope equipped with a digital camera. Quantification of tube formation, including tube length and structural complexity, was performed using ImageJ software (Version 1.44p, NIH, USA). Tubulogenesis was evaluated by a scoring system previously established in earlier studies (29, 30). Specifically, 25 colonies per well were examined and scored from 0 to 4:

• 0 = no sprouting;

• 1 = sprouting without branching;

• 2 = branching (arborization) present;

• 3 = presence of anastomoses;

• 4 = formation of an intricate tubular network.

The total tubulogenesis score per well was the sum of scores from all colonies, with a maximum achievable score of 100.

### Preparation of conditioned medium (CM)

To prepare conditioned medium for wound healing experiments, cells were cultured in standard conditions and then switched to serum-free medium. After 24 hr of incubation, the supernatant was collected, centrifuged to eliminate cellular debris, filtered, and stored at -80 ^°^C until further use.

### Wound healing model in diabetic mice

C57BL/6 mice with streptozotocin-induced diabetes were anesthetized using ketamine (100 mg/kg, i.m.) and xylazine (10 mg/kg, i.m.). A circular full-thickness excisional wound (~2 cm² or ~400 mm²) was created on the dorsal surface. After recovery, mice were housed individually in sanitized cages and assigned to one of two treatment groups:

• Group I (Control): Received peri-wound injections of conditioned medium derived from diabetic EPCs.

• Group II (EPC+DFO): Treated with conditioned medium from diabetic EPCs exposed to 80 µM DFO.

Wound areas were photographed on days 0, 5, 10, and 15 post-injuries. The percentage of wound closure was calculated using the following formula:

Wound Contraction (%) = [(Initial Area – Final Area) / Initial Area] × 100.

### VEGF quantification by ELISA

VEGF protein levels were determined using a commercial ELISA kit (Cat No: MMV00, R&D Systems, USA) according to the manufacturer’s instructions. Tissue lysates were prepared by rinsing wound tissues in PBS, mincing, homogenizing, and then incubating with RIPA buffer containing protease inhibitors. After centrifugation to remove debris, the supernatant was used in the ELISA. The standard steps of coating, blocking, sample and antibody addition, and substrate detection were followed to quantify VEGF levels.

### Western blot analysis

Wound granulation tissue lysates were processed for western blotting, adapted from previously reported protocols (29). Tissue or cell lysates were combined with 2× Laemmli buffer, boiled for 5 minutes, and 20 μg of protein was loaded onto SDS-PAGE gels. After electrophoresis, proteins were transferred to 0.2 μm PVDF membranes. Membranes were blocked for 1 hour in 3% non-fat milk in TBST (0.1% Tween-20 in TBS), and then probed with primary antibodies targeting Tie2, phospho-Tie2, HIF-1α, Ang1, Ang2, VEGF, SDF-1α, and β-actin (used as a loading control) for one hour at room temperature.

Following primary incubation, membranes were washed and incubated with HRP-conjugated secondary antibodies (e.g., sheep anti-rabbit IgG-HRP) for 1 hr. Protein bands were visualized using enhanced chemiluminescence (ECL) for 1-2 min. Densitometric quantification was conducted using ImageJ (Version 1.44, NIH, USA), with each target protein normalized to its corresponding β-actin band. Band intensities were compared between experimental groups based on previously described methodology.

### Statistical analysis

All results are presented as mean±standard deviation (SD). Prior to analysis, data were assessed for normal distribution and equality of variances. One-way analysis of variance (ANOVA) was applied to evaluate differences among multiple groups, followed by Tukey’s *post hoc* test for pairwise comparisons. For direct comparisons between two groups, the student’s t-test was employed.

## Results

### Morphological and cytotoxic effects of DFO on EPCs

**Figure 1:**
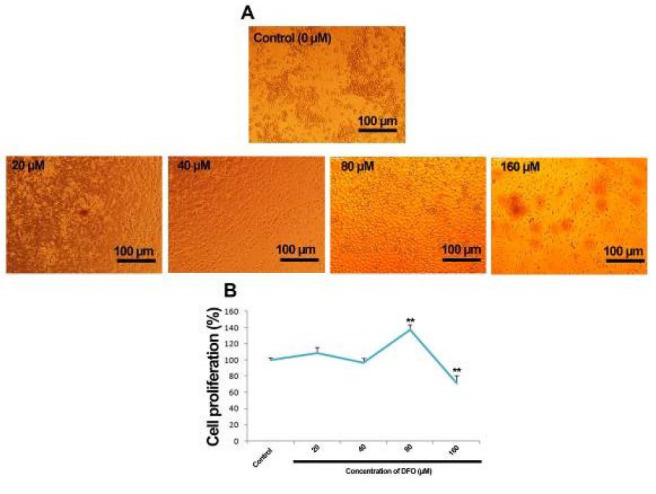
Effects of DFO on morphology and Proliferation of EPCs

To evaluate the impact of DFO on the morphology of EPCs, cells were exposed to increasing concentrations (20, 40, 80, and 160 µM) for 24 hr. Morphological observations revealed that treatment with 80 µM DFO led to the appearance of cobblestone-shaped EPCs with occasional spindle-like or fusiform cells on fibronectin-coated plates as early as seven days post-seeding (Figure 1A).

To assess cytotoxicity, the MTT assay was employed. Cell viability was interpreted as proliferative if ≥100% and cytotoxic if <100% (Figure 1B). Results indicated that 80 µM DFO enhanced cell growth, while 40 and 160 µM concentrations showed no significant impact. Based on these findings, 80 µM was selected for all further experiments.

### Immunophenotypic characterization of EPCs with and without DFO

**Figure 2:**
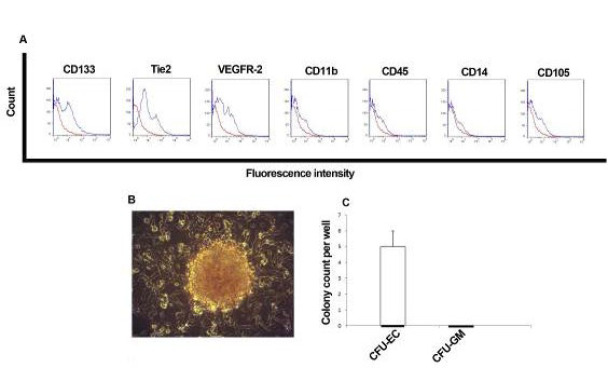
EPCs characteristics. Flow cytometric analysis of EPCs for CD133, Tie2, VEGFR-2, CD11b, CD45, CD11b, and CD105 (A). Outgrowth of colony-forming units of endothelial cells (CFU-EC) on the methylcellulose medium containing VEGF and GM-CSF after seeding of EPCs (B). Number of CFU-EC developed by EPCs on the methylcellulose medium in each group (C)

EPCs: Endothelial progenitor cells; VEGF: Vascular endothelial growth factor

Consistent with established markers and previous studies (31, 32, 10), EPCs were defined by the expression of VEGFR-2, CD133, and Tie2. Flow cytometry confirmed that cells expanded on fibronectin lacked expression of hematopoietic and myeloid lineage markers such as CD11b, CD14, CD105, and CD45 (Figure 2A). Furthermore, when seeded in methylcellulose medium supplemented with VEGF and GM-CSF, cells formed endothelial colony-forming units (CFU-EC) with a spindle-shaped appearance. No granulocyte-macrophage colonies (CFU-GM) were observed, further supporting the endothelial identity of these cells (Figure 2B, C).

**Figure 3:**
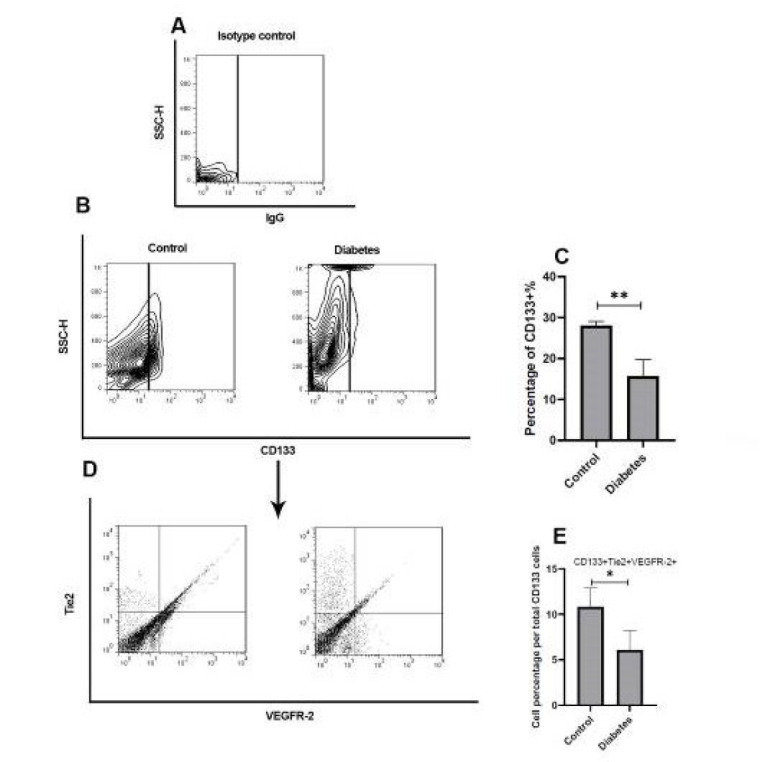
Gating strategy for analyzing EPCs by flow cytometry

Flow cytometry also revealed a significantly reduced proportion of CD133⁺/Tie2⁺/VEGFR-2⁺ cells in diabetic mice compared to non-diabetic controls (*P*<0.01)(Figure 3A, E).

### Clonogenic and proliferative capacity of EPCs post-DFO treatment

**Figure 4:**
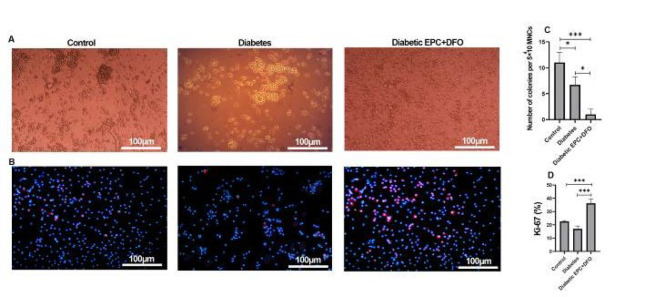
EPC colony-forming units. Colony formation of cells seeded on fibronectin after 7 days (A). Images represent images of Ki-67 staining of EPCs in the presence and absence of DFO (B). Several small colonies formed by EPCs in the presence and absence of DFO (C) and the percentage of Ki-67-positive cells (D).

Colony-forming capacity was monitored daily for EPCs isolated from both diabetic and non-diabetic animals (Figure 4A). Interestingly, EPCs from diabetic mice treated with PBS formed more colonies than those treated with DFO (Figure 4C), indicating a DFO-induced reduction in clonogenic potential.

To assess proliferative activity, Ki-67 immunostaining was performed. EPCs treated with DFO showed significantly higher Ki-67 expression levels than untreated controls, suggesting increased proliferation (Figure 4B, D).

### Effect of DFO on vasculogenic capacity of EPCs

**Figure 5:**
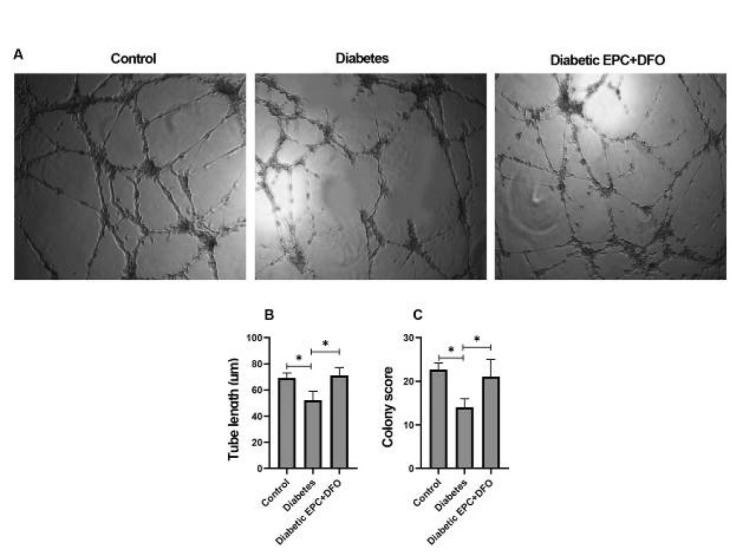
Matrigel tube formation assay in EPCs pre-cultured on fibronectin

The Matrigel tube formation assay was employed to evaluate the angiogenic potential of EPCs after DFO exposure. EPCs cultured for seven days from both diabetic and non-diabetic mice formed tube-like networks when pretreated with DFO, whereas EPCs from diabetic mice without DFO treatment failed to organize into capillary-like structures (Figure 5A). DFO-treated diabetic EPCs displayed a marked improvement in tube formation compared to untreated diabetic cells (Figure 5B, C).

### Wound healing potential of EPC-derived conditioned medium

**Figure 6:**
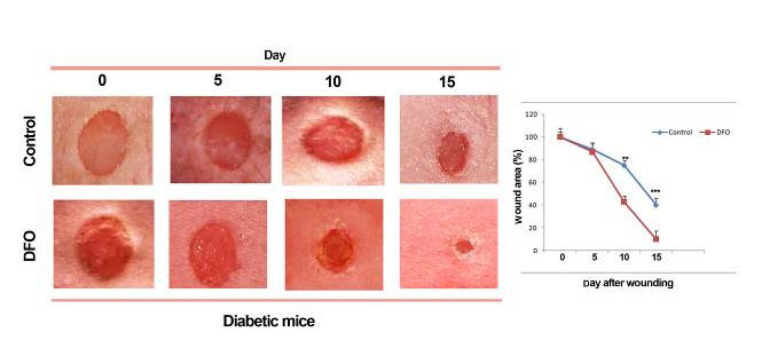
Wound healing progression was evaluated in diabetic C57BL/6 mice following administration of conditioned medium derived from endothelial progenitor cells (EPCs)

To assess the paracrine effects of EPCs, conditioned media (CM) from DFO-treated and untreated EPCs were applied in a wound healing model. Mice receiving CM from DFO-exposed EPCs exhibited significantly greater wound contraction, particularly on days 10 and 15 post-injury (*P*<0.01), compared to mice treated with CM from untreated EPCs (Figure 6).

### VEGF expression assessment via ELISA

**Figure 7:**
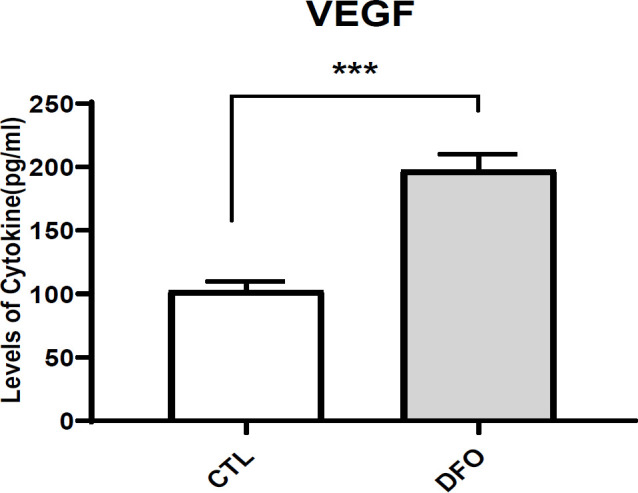
VEGF in the protein lysate of diabetic mice wound tissues

VEGF protein levels in wound tissues were quantified using ELISA. The analysis revealed significantly elevated VEGF concentrations in tissues from mice treated with CM derived from DFO-preconditioned EPCs compared to those treated with control CM (Figure 7).

### Protein expression analysis by Western blot

**Figure 8 (A-H):**
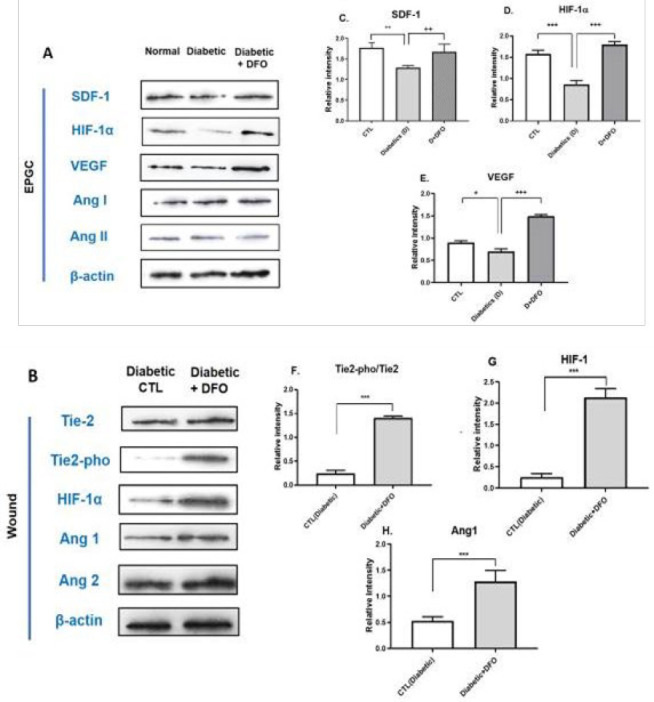
Western blot analysis of EPCs and wound tissue with or without DFO treatment

Western blotting was conducted to evaluate the expression of key angiogenic and signaling proteins. EPCs isolated from diabetic and non-diabetic mice, along with DFO-treated diabetic EPCs, were analyzed for VEGF, SDF-1α, Ang-1, Ang-2, and HIF-1α expression (Figure 8A). In parallel, wound tissues collected 15 days after CM injection were assessed for the levels of Tie2, phosphorylated Tie2 (Pho-Tie2), Ang1, Ang2, and HIF-1α (Figure 8B).

Results demonstrated that DFO treatment significantly increased the expression of VEGF, SDF-1α, and HIF-1α in EPCs (Figure 8C, D). In wound tissue, CM from DFO-treated EPCs led to a notable up-regulation of Pho-Tie2/Tie2, Ang1, and HIF-1α levels, indicating enhanced activation of pro-angiogenic pathways (Figure 8F, H).

## Discussion

Endothelial progenitor cell (EPC)-based therapies have gained considerable attention for their ability to stimulate neovascularization in ischemic tissues (33). However, the functionality of autologous EPCs can be compromised in pathological conditions that affect the vasculature, thus limiting their therapeutic effectiveness (34). To overcome this, strategies that restore or enhance the angiogenic properties of EPCs before transplantation are essential. Among these, hypoxic preconditioning has emerged as a viable method to increase cell survival and angiogenic capacity post-implantation (16, 35). Alternatively, the use of hypoxia-mimetic agents like DFO offers a practical and cost-effective approach to replicate the cellular effects of hypoxia without the need for specialized equipment (17).

This study investigated the impact of DFO on several biological characteristics of EPCs, including their proliferative capacity, ability to form capillary-like structures (tubulogenesis), differentiation potential, and wound healing efficacy. Findings demonstrated that DFO significantly enhanced EPC proliferation, differentiation, and tubulogenic potential while also improving their ability to promote tissue repair. Interestingly, DFO treatment resulted in reduced clonogenicity, potentially indicating a shift toward a more differentiated endothelial phenotype. Previous research has shown that iron chelators can affect cellular proliferation in different cell types, such as hepatoma cells (36) and adipose-derived stem cells (9). In the current work, 80 µM DFO effectively promoted EPC expansion *in vitro*.

Diabetes-induced hyperglycemia is known to impair angiogenesis, leading to poor vascular regeneration and delayed wound healing (37,9). Here, we demonstrated that conditioned medium derived from DFO-pretreated EPCs significantly accelerated wound closure in streptozotocin (STZ)-induced diabetic mice. This effect appears to be mediated through the stabilization of hypoxia-inducible factor 1-alpha (HIF-1α), a transcription factor central to the hypoxia response. HIF-1α promotes the transcription of angiogenic genes, including vascular endothelial growth factor (VEGF), which in turn stimulates endothelial proliferation and migration necessary for new blood vessel formation.

Moreover, our data revealed that DFO increased the expression of Angiopoietin-1 (Ang-1), a ligand that binds to the Tie2 receptor on endothelial cells, facilitating vascular stabilization and maturation. This dual mechanism—activation of HIF-1α/VEGF and Ang-1/Tie2 signalling—supports DFO’s role in both initiating and stabilizing angiogenesis. The observed increase in VEGF levels after DFO treatment was consistent with this mechanism. Additionally, a significant reduction in the proportion of CD133⁺ cells following DFO exposure suggests that DFO promotes endothelial lineage commitment, which is likely beneficial for wound repair.

Previous studies have reported enhanced neovascularization in ischemic tissues after DFO administration, associated with up-regulation of VEGF and endothelial marker CD31 (20, 38). DFO exerts its effects by inhibiting prolyl hydroxylases, thereby stabilizing HIF-1α even under normoxic conditions (39). HIF-1 activation leads to increased expression of multiple pro-angiogenic genes, such as Tie2, VEGF, stromal cell-derived factor 1 (SDF-1), and fibroblast growth factor 2 (FGF-2)(39, 40, 41). In line with these findings, we observed up-regulation of VEGF and HIF-1α in wound tissue treated with DFO-conditioned medium.

Additional literature supports the notion that HIF pathway activation by DFO can improve vascular development in various contexts, including lung tissue regeneration in bronchopulmonary dysplasia models (42). Furthermore, HIF-2α, another member of the HIF family, is known to regulate Tie2 expression in endothelial cells, further contributing to vessel formation and stability (41, 43).

Nevertheless, it is essential to note that prolonged DFO exposure may lead to EPC senescence, potentially impairing their regenerative capacity (44). Despite this limitation, the angiogenic and antioxidant actions of DFO position it as a promising pharmacological agent for enhancing regenerative therapies, particularly in the context of chronic wounds and bone repair (45).

Re-establishing a functional vasculature in injured tissues relies heavily on angiogenic remodeling, a process in which EPCs are recruited from the bone marrow to the ischemic site (22, 46). Our prior work demonstrated that Ang-1/Tie2 signalling promotes mature endothelial-like behavior in EPCs (47). In this study, we extend these findings by showing that DFO’s regenerative effects are, at least in part, mediated through the Ang-1/Tie2 pathway. Interestingly, VEGF is also known to regulate Tie2 signalling via phosphoinositide 3-kinase/Akt pathways, facilitating receptor shedding, which is a key step in endothelial cell activation and migration (48). Our results are in agreement with reports suggesting that DFO enhances Ang-1 expression (49), further implicating this axis in vascular regeneration.

Morphology (A) and proliferation rate of EPC after exposure to different concentrations of DFO (B). Data are presented as mean±SD. Three independent experiments were performed in triplicate. **P<0.01 versus control (Student’s t-test)

DFO: Deferoxamine; EPCs: Endothelial progenitor cells

Isotype control (A), CD133+cells were first gated (B) and then sequentially analyzed for VEGFR2 and Tie2 (C) for each group. Percentage of CD133+VEGFR2+Tie2+EPCs per total mononuclear cell fraction (C). Data are expressed as mean±SD (n=6, each group). *P<0.05 **P<0.01. Student’s t-test

EPCs: Endothelial progenitor cells

Data are expressed as mean±SD, three independent experiments were performed. *P<0.05, ***P<0.001. (One-way ANOVA with Tukey *post hoc *test)

EPCs: Endothelial progenitor cells; DFO: Deferoxamine

Representative images depict the tubulogenesis of early EPCs with or without DFO treatment (A). Analysis of tube length (B), and colony tubulogenesis scoring (C) in early EPCs pre-expanded on fibronectin shows decreased angiogenic activity in untreated EPCs compared to those treated with DFO. Results are presented as mean±SD; experiments were conducted independently in triplicate, **P<0.01 (one-way ANOVA with Tukey *post hoc* test)

EPCs: Endothelial progenitor cells; DFO: Deferoxamine

Representative images of the wound sites were captured on days 0, 5, 10, and 15 post-injection with EPC-conditioned medium, either with or without DFO supplementation (A). Day 0 images correspond to the immediate post-injury state. The extent of wound closure was quantitatively assessed over time (B), with five mice included per group (n=5). Statistical comparisons at each time point between groups were performed using Student’s t-test

EPCs: Endothelial progenitor cells; DFO: Deferoxamine

Concentrations of VEGF in wounds of mice received the conditioned medium of EPCs treated with or without DFO 15 days after injection. Data are expressed as mean±SD (n=5 in each group). *P<0.05, **P<0.01, Student’s t-test

EPCs: Endothelial progenitor cells; VEGF: Vascular endothelial growth factor; DFO: Deferoxamine

Panels A and B display representative western blot bands. Relative protein expression levels of VEGF (C), SDF-1 (D), and HIF-1α (E) in EPCs, and Tie2-phosphorylation normalized to total Tie2 (F), HIF-1α (G), and Angiopoietin-1 (H) in wound tissue are shown. Protein expression was quantified by calculating the area under the curve (AUC) for each band, normalized to the AUC of the corresponding β-actin control. Data are presented as mean±SD from three independent experiments conducted in triplicate. Statistical analysis was performed using one-way ANOVA followed by Tukey’s post hoc test (*P<0.05)

Western blot analysis of Tie2 and phosphorylated Tie2

Panel A shows representative blot images, and panel B displays the quantification of phosphorylated Tie2 normalized to total Tie2. Densitometric values were normalized to β-actin, and statistical differences between groups were assessed using Student’s t-test. Data are presented as mean±SD from three independent triplicate experiments (*P<0.05)

EPCs: Endothelial progenitor cells; DFO: Deferoxamine; VEGF: Vascular endothelial growth factor

## Conclusion

This study presents pharmacological preconditioning with DFO as a viable strategy to restore the angiogenic potential of EPCs from diabetic individuals. DFO not only promotes EPC proliferation and differentiation but also revives their compromised function. The dual activation of the HIF-1α/VEGF and Ang-1/Tie2 pathways plays a pivotal role in improving both the initiation and stabilization phases of neovascularization, making DFO a promising adjunct in regenerative medicine and wound healing applications.

## Declaration

The authors affirm that ChatGPT (OpenAI) was used solely for the purpose of improving the language and grammar of the manuscript. It was not used to generate scientific content, interpret data, or draw conclusions. All intellectual and analytical content is the original work of the authors.
